# In-line mammalian cell concentration measurement with a Bio Cell Vitality Analyzer

**DOI:** 10.1186/1753-6561-9-S9-P64

**Published:** 2015-12-14

**Authors:** Patrick Steger, Dirk Lütkemeyer

**Affiliations:** 1 Institute for Protein Characterisation, Faculty of Engineering and Mathematics, University of Applied Science Bielefeld, Bielefeld, Germany

## Background

Cell cultivations in a 30L bioreactor were in situ and on-line monitored using the Bio Cell Vitality Analyzer (BCVA, Sequip S+E, Düsseldorf, Germany).

Based on the technique of optical reflectance measurement the BCVA is able to detect mammalian cells by combining reflectance measurement with a three dimensional moving focus point and a time of flight measurement.

The aim of the experiments was to verify the process usability of the BCVA in bioreactor cultivation. Furthermore the BCVA shall be compared with the two already established Casy and Cedex devices (both Roche, Basel Switzerland). In contrast to the Cedex and Casy where a sample preparation is necessary the BCVA is able to measure the cells in situ. Moreover the particle distribution over time can be plotted.

## Materials and Methods

The dilution series and the cultivation have been performed as follows.

Cells: Recombinant Chinese Hamster Ovary Cells (CHO) producing a monoclonal antibody.

Media: MAM-PF7, Bioconcept, Allschwil, Switzerland. The medium is serum and protein free and chemically defined.

Feeds: If necessary the cells have been fed with a glucose and glutamine solution.

Measurement setup: For the dilution series a 1 liter beaker with magnetic stirrer and BCVA fixed on a stand with the measurement window positioned against the liquid stream.

Bioreactor: C-DCU 30 liter stainless steel bioreactor, Sartorius Stedim Biotech, Göttingen, Germany.

Parameter control: The agitation of the culture has been performed with two 2-blade impeller at 100 rpm. For pH adjustment CO2 has been added by the overlay stream. The Oxygen-concentration has been controlled via aeration of air/pure oxygen through the ring-sparger.

## Results

To prove the ability of the BCVA to measure CHO-cells a dilution series has been performed. In table 1 the resulting cell concentrations measured by Casy and Cedex are shown and compared with the measured particle number of the BCVA. The resulting data show nearly the same progression in a cell concentration range of 2·105 and 60 ·105 cells per milliliter. Furthermore a small offset of the BCVA to the other two curves exists which can be deleted by performing a calibration. By plotting the resulting cell concentrations of the Cedex and Casy versus the BCVA particle counts the resulting points fit to a quadratic regression. Based on the fact that the BCVA was measuring in undiluted cell suspension this quadratic regression proves the influence of saturation effects on the measurement.

**Table 1:** Measurement results for the three different systems Casy, Cedex and BCVA performing a dilution series

Dilution step	Casy [x10^5^Cells/ml]	Cedex [x10^5^Cells/ml]	BCVA [Quantity]
1	126,7	134,87	3513,23
2		98,32	2918,75
3	74,79	75,22	2464,52
4	57,39	62,73	2317,81
5	31,33	29,81	1436,36
6	12,76	13,54	760,11
7	6,07	6,03	389,03
8	4,29	3,72	237,59
9	2,87	2,12	165,69
10	1,76	1,8	129,62

As a side effect of our investigations we realized that the BCVA is able to measure an upcoming bacterial contamination earlier compared for example to the drop of the oxygen concentration. With the ability to on-line measure particle diameters and particle numbers the BCVA is able to clearly distinguish a bacterial from a CHO-cell growth. Furthermore this signal gives the operator a strong hint that the culture is free of a contamination.

**Figure 1:**
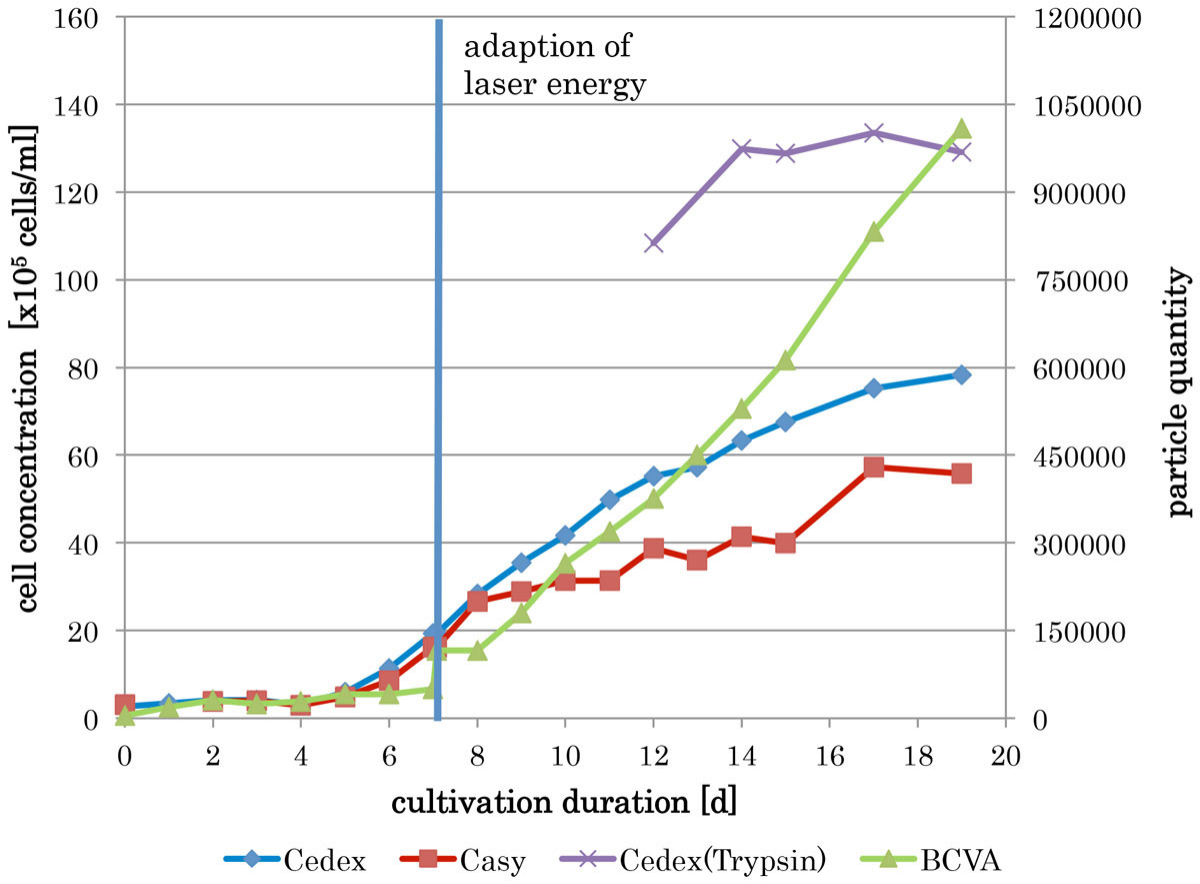
Growth curve measured by Casy, Cedex and BCVA during a cultivation in a 30 litre bioreactor

During a 19 day cultivation the ability of the BCVA to measure CHO cells in a culture was tested. The observation of the cultivated CHO-cells (figure 1) during the cultivation was possible to a certain extend only. During the cultivation various effects influenced the measurement results of all three systems. The greatest problem was an aggregation of the cells which prevents the correct measurement of the cell number by every system. To get the real concentration of the cultivated cells the cells have been trypsinized. At the beginning of the growth phase the laser beam intensity of the BCVA has to be adapted to the culture conditions. After the adaption it shows a similar progression compared with the Cedex for nearly 7 days. When the aggregates start to form both systems the Cedex and the BCVA starts to show different results caused by their ability to resolute aggregates. Since the Casy is not able to distinguish precisely aggregates from single cells, the measured cell concentration is to low compared to the other cell count techniques.

Remarkably, in the stationary phase of the cultivation the formed cell aggregates start to disintegrate although the viability is still high in this late phase of the cultivation. The BCVA is able to illustrate this phenomenon more exactly, whereas the Casy and the Cedex are not.

The histograms generated by the BCVA shows only a similarity in the size distribution compared to the Casy. For particle diameters above 16 micrometer unspecific reflections influenced the measurements results.

## Conclusions

The analyzer is able to observe cellular growth and rising cell concentrations in situ and on-line. Moreover it is able to detect an upcoming contamination precisely and early.

In addition to this the BCVA can be used to automate processes for the nutrient addition or the harvesting. With the setting of a cell concentration threshold the on-line system can initiate such processes in the absence of an operator.

Furthermore optical parameters like the obscurance factor can be used for the addition of essential nutrients.

During the experiments limitations in the use of the BCVA has been found. The use of the BCVA for the generation of a histogram is only possible under certain circumstances. The presence of particles which reflect the laser beam in unspecific manner makes it difficult to determine cellular diameters. The reduction of this unspecific reflectance should be the aim of further studies.

## Acknowledgements

The supply of the BCVA and the assistance during the cultivations by Sequip S+E GmbH is greatly acknowledged.

